# Supplementary material for: Dietary and nutritional interventions in children with cerebral palsy: A systematic literature review
Source: PLoS One. 2022 Jul 22;17(7):e0271993. doi: 10.1371/journal.pone.0271993 (PMC9307182; doi:10.1371/journal.pone.0271993)
Supplement: S2 Appendix — (PDF) [file pone.0271993.s004.pdf]

National Institute for Health Research

University of York
Centre for Reviews and Dissemination

Systematic review

1. * Review title.

Give the title of the review in English

Nutritional and dietary interventions in children with cerebral palsy: systematic literature review and potential meta-analysis.

2. Original language title.

For reviews in languages other than English, give the title in the original language. This will be displayed with the English language title.

Intervenções nutricionais e dietéticas em crianças com paralisia cerebral: revisão sistemática de literatura com potencial metanálise.

3. * Anticipated or actual start date.

Give the date the systematic review started or is expected to start.

03/02/2020

4. * Anticipated completion date.

Give the date by which the review is expected to be completed.

30/07/2021

5. * Stage of review at time of this submission.

Tick the boxes to show which review tasks have been started and which have been completed. Update this field each time any amendments are made to a published record.

Reviews that have started data extraction (at the time of initial submission) are not eligible for inclusion in PROSPERO. If there is later evidence that incorrect status and/or completion date has been supplied, the published PROSPERO record will be marked as retracted.

This field uses answers to initial screening questions. It cannot be edited until after registration.

The review has not yet started: Yes

Review stage	Started	Completed
Preliminary searches	No	No
Piloting of the study selection process	No	No
Formal screening of search results against eligibility criteria	No	No
Data extraction	No	No
Risk of bias (quality) assessment	No	No
Data analysis	No	No

Provide any other relevant information about the stage of the review here.

6. * Named contact.

The named contact is the guarantor for the accuracy of the information in the register record. This may be any member of the review team.

Dr Fernanda Rebelo

Email salutation (e.g. "Dr Smith" or "Joanne") for correspondence:

Dr Rebelo

7. * Named contact email.

Give the electronic email address of the named contact.

frebelos@gmail.com

8. Named contact address

Give the full institutional/organisational postal address for the named contact.

Unidade de Pesquisa ClÃnica, Instituto Fernandes Figueira. \nAvenida Rui Barbosa, 716, Flamengo. Rio de Janeiro\/ RJ, Brazil. \nCEP: 22250-020

9. Named contact phone number.

Give the telephone number for the named contact, including international dialling code.

0552125541912

10. * Organisational affiliation of the review.

Full title of the organisational affiliations for this review and website address if available. This field may be completed as 'None' if the review is not affiliated to any organisation.

National Institute of Women, Children and Adolescents' Health Fernandes Figueira, Oswaldo Cruz Foundation

Organisation web address:

11. * Review team members and their organisational affiliations.

Give the personal details and the organisational affiliations of each member of the review team. Affiliation refers to groups or organisations to which review team members belong. **NOTE: email and country now MUST be entered for each person, unless you are amending a published record.**

Dr Fernanda Rebelo. National Institute of Women, Children and Adolescents' Health Fernandes Figueira, Oswaldo Cruz Foundation
Dr Maria Elisabeth Moreira. National Institute of Women, Children and Adolescents' Health Fernandes Figueira, Oswaldo Cruz Foundation
Dr Saint-Clair Junior. National Institute of Women, Children and Adolescents' Health Fernandes Figueira, Oswaldo Cruz Foundation
Dr Vania Fonseca. National Institute of Women, Children and Adolescents' Health Fernandes Figueira, Oswaldo Cruz Foundation
Dr Maria Dalva Baker. National Institute of Women, Children and Adolescents' Health Fernandes Figueira, Oswaldo Cruz Foundation
Dr Fernanda Soares. National Institute of Women, Children and Adolescents' Health Fernandes Figueira, Oswaldo Cruz Foundation
Dr Daniele Marano. National Institute of Women, Children and Adolescents' Health Fernandes Figueira, Oswaldo Cruz Foundation
Dr Ana Carolina Costa. National Institute of Women, Children and Adolescents' Health Fernandes Figueira, Oswaldo Cruz Foundation
Sylvia Nehab. National Institute of Women, Children and Adolescents' Health Fernandes Figueira, Oswaldo Cruz Foundation
Teresa Miglioli. National Institute of Women, Children and Adolescents' Health Fernandes Figueira, Oswaldo Cruz Foundation
Isabela Mansur. National Institute of Women, Children and Adolescents' Health Fernandes Figueira, Oswaldo Cruz Foundation

12. * Funding sources/sponsors.

Details of the individuals, organizations, groups, companies or other legal entities who have funded or sponsored the review.

Brazilian National Council for Scientific and Technological Development (CNPq)

Grant number(s)

State the funder, grant or award number and the date of award

process number: 442551/2019-3

13. * Conflicts of interest.

List actual or perceived conflicts of interest (financial or academic).

None

14. Collaborators.

Give the name and affiliation of any individuals or organisations who are working on the review but who are not listed as review team members. **NOTE: email and country must be completed for each person, unless you are amending a published record.**

15. * Review question.

State the review question(s) clearly and precisely. It may be appropriate to break very broad questions down into a series of related more specific questions. Questions may be framed or refined using PI(E)COS or similar where relevant.

1. What is the effect of nutritional and dietary interventions on clinical and nutritional aspects, neurodevelopment and seizure control in children with cerebral palsy?

2. What are the reporting characteristics (population and methods) and quality of the studies that aimed to assess the effect of nutritional and dietary interventions in children with cerebral palsy?

3. What are the nutritional and dietary interventions that have already been studied in the therapy of children with cerebral palsy?

4. What are the main benefits (outcomes) of nutritional and dietary interventions for children with cerebral palsy?

5. What are the main limitations of the studies found and what are the next steps for the advancement of knowledge in this area?

16. * Searches.

State the sources that will be searched (e.g. Medline). Give the search dates, and any restrictions (e.g. language or publication date). Do NOT enter the full search strategy (it may be provided as a link or attachment below.)

The search for studies will include automatic (online) and manual surveys. For the online search, the databases LILACS (via Bireme), MEDLINE (via PubMed), Web of Science, Embase and Scopus (via the Capes periodical portal) will be consulted to identify publications that meet the eligibility criteria. The manual search for studies will be carried out, from the list of references of the selected articles.
In order to minimize publication bias, studies will also be evaluated, even if not published and/or indexed, identified by ClinicalTrials.gov, National Technical Information Services, Brazilian Digital Library of Theses and Dissertations, ProQuest Dissertations and Theses Database, Open Gray, Trove, BVS and CENTRAL (The Cochrane Central Register of Controlled Trials The Cochrane Library).

The following keywords or MeSH terms will be considered as descriptors: (“cerebral palsy” OR “cerebral pals*”) AND (child* OR “child, preschool” OR “child, school”) AND (nutrients OR nutrition OR diet* OR “nutrition therapy” OR “diet therapy” OR “dietary supplements”) AND (“clinical trial” OR “experimental study” OR randomized).

The search will be adapted according to the database being searched. Additionally, in place of the terms that identify the study design, the high-sensitivity strategy for identifying randomized clinical trials developed by Cochrane will be used for searches on PubMed and EMBASE.

The search is scheduled to begin in April 2020. There will be no language and year of publication restrictions. The searches will be run again and the results updated before statistical analysis be performed.

17. URL to search strategy.

Upload a file with your search strategy, or an example of a search strategy for a specific database, (including the keywords) in pdf or word format. In doing so you are consenting to the file being made publicly accessible. Or provide a URL or link to the strategy. Do NOT provide links to your search **results**.

Alternatively, upload your search strategy to CRD in pdf format. Please note that by doing so you are consenting to the file being made publicly accessible.

Do not make this file publicly available until the review is complete

National Institute for Health Research

18. * Condition or domain being studied.

Give a short description of the disease, condition or healthcare domain being studied in your systematic review.

Cerebral palsy, also known as chronic non-progressive encephalopathy, is an extremely serious, disabling brain injury, with repercussions that are not limited to the individual himself, affecting his family and society. It comprises a group of permanent and non-progressive disorders of movement and posture secondary to injury, dysfunction or damage to the development of the fetal or infant brain. These motor disorders are often accompanied by sensory changes, in perception, cognition, communication, behavior, epileptic seizures, secondary musculoskeletal problems and important comorbidities, such as behavioral, growth and gastrointestinal disorders.

19. * Participants/population.

Specify the participants or populations being studied in the review. The preferred format includes details of both inclusion and exclusion criteria.

Children of preschool or school age (2 to 12 years) with cerebral palsy;

20. * Intervention(s), exposure(s).

Give full and clear descriptions or definitions of the interventions or the exposures to be reviewed. The preferred format includes details of both inclusion and exclusion criteria.

Nutritional and dietary intervention, including, but not limited to:

Additive or restrictive dietary modifications,

Macro and / or micronutrient supplementation.

21. * Comparator(s)/control.

Where relevant, give details of the alternatives against which the intervention/exposure will be compared (e.g. another intervention or a non-exposed control group). The preferred format includes details of both inclusion and exclusion criteria.

Group of children with cerebral palsy who did not receive the nutritional/dietary intervention under investigation.

22. * Types of study to be included.

Give details of the study designs (e.g. RCT) that are eligible for inclusion in the review. The preferred format includes both inclusion and exclusion criteria. If there are no restrictions on the types of study, this should be stated.

Controlled and randomized clinical trials will be included. Other intervention studies (uncontrolled and quasi-experimental clinical trials), observational studies (case-control, cross-sectional, case series) and reviews (integrative, narrative, letters) will be excluded.

23. Context.

Give summary details of the setting or other relevant characteristics, which help define the inclusion or exclusion criteria.

We will include all studies meeting the inclusion/exclusion criteria, regardless of setting or context.

24. * Main outcome(s).

Give the pre-specified main (most important) outcomes of the review, including details of how the outcome is defined and measured and when these measurement are made, if these are part of the review inclusion criteria.

We will broadly include studies that evaluated short and long-term health outcomes. The main outcomes of interest are:

1. Clinical aspects, including but not limited to:

• Anemia

• Changes in muscle tone

• Constipation

• Difficulty sleeping and sleeping irregularly

• Gastrostomy

• Sialorrhea (drool)

2. Nutritional aspects, including but not limited to:

• Eating difficulties: impairment of the oral motor phase, choking, nausea, difficulty in transporting the bolus, prolonged or interrupted meals and gastroesophageal reflux

• Nutritional status, measured by: weight, height, BMI, arm perimeter, tricipital skinfold, subscapular skinfold, muscular area of the arm, fatty area of the arm, and others

• Swallowing disorders

3. Neurodevelopment, including but not limited to:

• Adaptive behavior

• Attention span

• Cognitive evaluation

• Functional assessment for vision and hearing

• Functional independence

• Graphomotor abilities

• Language skills

• Motor skills

• Neurologic examination

• Social-emotional skills

4. Convulsive control

Measures of effect

Please specify the effect measure(s) for you main outcome(s) e.g. relative risks, odds ratios, risk difference, and/or 'number needed to treat.

Due to the wide variety of possible interventions and outcomes in the proposed review, it is expected that different measures of effect will be found. All will be recorded and reported as a result of the SLR and only those that can be combined will be used in the meta-analysis.

25. * Additional outcome(s).

List the pre-specified additional outcomes of the review, with a similar level of detail to that required for main outcomes. Where there are no additional outcomes please state 'None' or 'Not applicable' as appropriate to the review

None

Measures of effect

Please specify the effect measure(s) for you additional outcome(s) e.g. relative risks, odds ratios, risk difference, and/or 'number needed to treat.

None

26. * Data extraction (selection and coding).

Describe how studies will be selected for inclusion. State what data will be extracted or obtained. State how this will be done and recorded.

The search results in all databases will be inserted in the Zotero software to remove duplicates. Subsequently, studies will be uploaded to Covidence, where the next stages of the selection will be carried out and registered. The first stage of the selection will consist of reading the title and abstract to identify and remove studies that are clearly irrelevant to the SLR objective. As a second stage, all studies selected in the first filter will be read in full to apply the eligibility criteria. The entire selection process will be done in duplicate by independent and blind reviewers. At the end of every stage of the selection, the results of each reviewer will be compared and the possible conflicts will be resolved by consensus among the project members.

The data of the included studies will be extracted using a standardized and pre-tested form, with fields structured in order to collect the relevant data for the SLR and meta-analysis in an objective way. Extracted information will include: author, year of publication, country, setting, recruitment method, randomization method, sample size, population characteristics (age, sex, nutritional status, etc.), detailed description of the intervention and outcomes, statistical analysis methods and main results. If necessary, missing data will be requested by email to the authors.

Data extraction will be performed by two independent researchers and possible disagreements will be resolved by consensus. The data obtained will be entered into Excel® spreadsheets in which the tables of the systematic review will be prepared. Later, these data will be exported to the Stata 16.0 software, where the meta-analysis will be carried out.

27. * Risk of bias (quality) assessment.

State which characteristics of the studies will be assessed and/or any formal risk of bias/quality assessment tools that will be used.

The methodological quality of the included studies will be carried out using the Version 2 of the Cochrane risk-of-bias tool for randomized trials (RoB 2). This tool takes into account the following characteristics: randomization process, assignment to the intervention, adhesion to the intervention, missing outcome data, measurement of the outcome and results report. Risk of bias will be classified as Low, High or Some concerns.

The studies will be evaluated by two researchers, independently. To resolve any disagreements in the scoring of articles, the opinion of a third researcher will be consulted.

28. * Strategy for data synthesis.

Describe the methods you plan to use to synthesise data. This **must not be generic text** but should be **specific to your review** and describe how the proposed approach will be applied to your data. If meta-analysis is planned, describe the models to be used, methods to explore statistical heterogeneity, and software package to be used.

We will provide a qualitative synthesis of the findings from the included articles, formatted as a table structured around the study characteristics (author, year of publication, place of performance, recruitment method, randomization method, sample size, population characteristics, intervention, outcomes, methods of statistical analysis and results).

To perform a meta-analysis it will be necessary a minimum of two studies investigating similar intervention and outcome. Depending on how the association of interest was quantified in each study group (separate groups according to the type of brain injury and the type of nutritional/dietary intervention), one or more summary measures will be calculated. To analyze the effect of the intervention on continuous outcomes, weighted mean difference with a 95% confidence interval (CI) will be used. For dichotomous variables, the effect size of the intervention will be expressed as relative risk (RR) and its respective 95% CI.

The effect size will be estimated using the random effects model, weighted by the inverse of the variance. The results will be presented as Forest plots.

29. * Analysis of subgroups or subsets.

State any planned investigation of ‘subgroups’. Be clear and specific about which type of study or participant will be included in each group or covariate investigated. State the planned analytic approach.

The studies will be grouped according to the intervention performed and/or the investigated outcome. In the meta-analysis, if moderate to high heterogeneity is observed, it will be explored by subgroup analyzes, according to factors such as: age, economic development of the country where it was carried out, the methodological quality of each study individually and the duration of the intervention.

In addition, the [illegible] size of the combined effect will be assessed through a sensitivity analysis. For this, each study will be omitted, one by one, in successive stages. The impact of the components of the quality assessment of the included studies (for example, randomization, blinding, loss of follow-up, etc.) and the impact of studies with a high risk of bias on the

results of meta-analyzes will also be explored.

For further clarification, a meta-regression method may be used, considering, for example, the nutritional status of participants in the baseline, dosage and frequency of supplementation.

30. * Type and method of review.

Select the type of review, review method and health area from the lists below.

Type of review

Cost effectiveness
No

Diagnostic
No

Epidemiologic
No

Individual patient data (IPD) meta-analysis
No

Intervention
No

Living systematic review
No

Meta-analysis
No

Methodology
No

Narrative synthesis
No

Network meta-analysis
No

Pre-clinical
No

Prevention
No

Prognostic
No

Prospective meta-analysis (PMA)
No

Review of reviews
No

Service delivery
No

Synthesis of qualitative studies
No

Systematic review
Yes

Other
No

Health area of the review

Alcohol/substance misuse/abuse
No

Blood and immune system
No

Cancer
No

Cardiovascular
No

Care of the elderly
No

Child health
Yes

Complementary therapies
No

COVID-19
No

Crime and justice
No

Dental
No

Digestive system
No

Ear, nose and throat
No

Education
No

Endocrine and metabolic disorders
No

Eye disorders
No

General interest
No

Genetics
No

Health inequalities/health equity
No

Infections and infestations

No

International development
No

Mental health and behavioural conditions
No

Musculoskeletal
No

Neurological
No

Nursing
No

Obstetrics and gynaecology
No

Oral health
No

Palliative care
No

Perioperative care
No

Physiotherapy
No

Pregnancy and childbirth
No

Public health (including social determinants of health)
No

Rehabilitation
No

Respiratory disorders
No

Service delivery
No

Skin disorders
No

Social care
No

Surgery
No

Tropical Medicine
No

Urological
No

Wounds, injuries and accidents
No

Violence and abuse
No

31. Language.

Select each language individually to add it to the list below, use the bin icon to remove any added in error.
English
Portuguese-Brazil

There is an English language summary.

32. * Country.

Select the country in which the review is being carried out. For multi-national collaborations select all the countries involved.

Brazil

33. Other registration details.

Name any other organisation where the systematic review title or protocol is registered (e.g. Campbell, or The Joanna Briggs Institute) together with any unique identification number assigned by them. If extracted data will be stored and made available through a repository such as the Systematic Review Data Repository (SRDR), details and a link should be included here. If none, leave blank.

34. Reference and/or URL for published protocol.

If the protocol for this review is published provide details (authors, title and journal details, preferably in Vancouver format)

Add web link to the published protocol.

Or, upload your published protocol here in pdf format. Note that the upload will be publicly accessible.

No I do not make this file publicly available until the review is complete

Please note that the information required in the PROSPERO registration form must be completed in full even if access to a protocol is given.

35. Dissemination plans.

Do you intend to publish the review on completion?

Yes

Give brief details of plans for communicating review findings.?

In addition to the results publication in a high-impact and open access journal, the data will also be disseminated through the Good Clinical Practice Portal, which is an initiative of the National Institute of Health for Women, Children and Adolescents Fernandes Figueira (IFF), from the Oswaldo Cruz Foundation (Fiocruz), from the Brazilian Ministry of Health (MS), in order to expand the possibility of generating and disseminating the knowledge obtained for the implementation of health policies and programs based on the best available scientific evidence. Moreover, contact will be established with the Brazilian Society of Pediatrics, the National Council of Nutritionists and the Brazilian Association of Cerebral Palsy in order to disseminate relevant data from the

analyzes carried out on their websites, social networks and magazines.

36. Keywords.

Give words or phrases that best describe the review. Separate keywords with a semicolon or new line. Keywords help PROSPERO users find your review (keywords do not appear in the public record but are included in searches). Be as specific and precise as possible. Avoid acronyms and abbreviations unless these are in wide use.

Cerebral palsy, brain injury, nutritional therapy, randomized controlled trial, systematic literature review, meta-analysis.

37. Details of any existing review of the same topic by the same authors.

If you are registering an update of an existing review give details of the earlier versions and include a full bibliographic reference, if available.

38. * Current review status.

Update review status when the review is completed and when it is published.New registrations must be ongoing so this field is not editable for initial submission.
Please provide anticipated publication date

Review_Ongoing

39. Any additional information.

Provide any other information relevant to the registration of this review.

40. Details of final report/publication(s) or preprints if available.

Leave empty until publication details are available OR you have a link to a preprint (NOTE: this field is not editable for initial submission). List authors, title and journal details preferably in Vancouver format.

Give the link to the published review or preprint.
