# Supplementary material for: Dietary and nutritional interventions in children with cerebral palsy: A systematic literature review
Source: PLoS One. 2022 Jul 22;17(7):e0271993. doi: 10.1371/journal.pone.0271993 (PMC9307182; doi:10.1371/journal.pone.0271993)
Supplement: S3 Appendix — (DOCX) [file pone.0271993.s005.docx]

**S1 Appendix**

**SEARCH SUMMARY: DATABASES, SEARCH STRATEGIES AND NUMBER OF IDENTIFIED RECORDS**

| 1. Results of the search strategies in electronic databases for published articles. | | |
| --- | --- | --- |
| **Databases** | **Search strategies** | **Identified records** |
| LILACS/Bireme | #1 "cerebral palsy" OR “chronic non progressive encephalopathy” OR "parálisis cerebral" [Palavras]  #2 child OR children OR childhood OR ninos OR ninas [Palavras]  #3 nutrients OR nutrition OR diet OR "nutrition therapy" OR "diet therapy" OR "dietary supplements" OR nutricion OR dieta [Palavras]  #1 AND #2 AND #3 | 37 |
| Medline/Pubmed | #1 "Cerebral Palsy"[Mesh] OR “cerebral pals*” OR “chronic non progressive encephalopathy”  #2 "Child"[Mesh] OR "Child, Preschool"[Mesh] OR "Child*"  #3 "Nutrition Therapy"[Mesh] OR "Diet"[Mesh] OR "Nutrients"[Mesh] OR "Dietary Supplements"[Mesh] OR nutrients OR nutrition OR diet* OR “nutrition therapy” OR “diet therapy” OR “dietary supplements”  #4 ((randomized controlled trial[pt]) OR (controlled clinical trial[pt]) OR (randomized[tiab]) OR (placebo[tiab]) OR (drug therapy[sh]) OR (randomly[tiab]) OR (trial[tiab]) OR (groups[tiab])) NOT (animals[mh] NOT humans[mh])  #1 AND #2 AND #3 AND #4 | 133 |
| Web of Science | #1 ALL=("cerebral palsy" OR "chronic non progressive encephalopathy")  #2 ALL=(child OR children OR childhood)  #3 ALL=(nutrients OR nutrition OR diet OR "nutrition therapy" OR "diet therapy" OR "dietary supplements")  #4 ALL=("clinical trial" OR "experimental study" OR randomized OR randomly OR "controlled trial" OR placebo)  #1 AND #2 AND #3 AND #4 | 79 |
| Embase | #1 ALL=("cerebral palsy" OR "chronic non progressive encephalopathy")  #2 ALL=(child OR children OR childhood)  #3 ALL=(nutrients OR nutrition OR diet OR "nutrition therapy" OR "diet therapy" OR "dietary supplements")  #4 ALL=("clinical trial" OR "experimental study" OR randomized OR randomly OR "controlled trial" OR placebo)  #1 AND #2 AND #3 AND #4 AND ([embase]/lim NOT ([embase]/lim AND [medline]/lim)) | 35 |
| Scopus | TITLE-ABS-KEY ( ( "cerebral palsy" OR “chronic non progressive encephalopathy”) AND ( child OR children OR childhood) AND ( nutrients OR nutrition OR diet OR "nutrition therapy" OR "diet therapy" OR "dietary supplements" ) AND ( "clinical trial" OR "experimental study" OR randomized OR randomly OR "controlled trial" OR placebo) ) | 137 |
| **TOTAL** | | **421** |

* Search date: June 17th, 2021

| 1. Results of the search strategies for registers databases. | | |
| --- | --- | --- |
| **Databases** | **Search strategies** | **Identified records** |
| ClinicalTrials.gov | nutrients OR nutrition OR diet OR "nutrition therapy" OR "diet therapy" OR "dietary supplements" \| Completed Studies \| "cerebral palsy" OR “chronic non progressive encephalopathy” | 11 |
| Cochrane Central Register of Controlled Trials | "Cerebral Palsy" OR “cerebral pals*” OR “chronic non progressive encephalopathy” in Title Abstract Keyword AND child OR children OR childhood in Title Abstract Keyword AND nutrients OR nutrition OR diet OR "nutrition therapy" OR "diet therapy" OR "dietary supplements" in Title Abstract Keyword AND "clinical trial" OR "experimental study" OR randomized OR randomly OR "controlled trial" OR placebo in Title Abstract Keyword - (Word variations have been searched) | 51 |
| **TOTAL** | | **62** |

* Search date: June 17th, 2021

| 1. Results of the search strategies for gray literature. | | |
| --- | --- | --- |
| **Databases** | **Search strategies** | **Identified records** |
| Brazilian Digital Library of Theses and Dissertations | Todos os campos:"paralisia cerebral" E Todos os campos:nutric* OR diet* E Todos os campos:criança* OR infantil | 21 |
| ProQuest Dissertations and Theses Database | (AB,TI( "cerebral palsy" OR “chronic non progressive encephalopathy”) AND ( child OR children OR childhood) AND ( nutrients OR nutrition OR diet OR "nutrition therapy" OR "diet therapy" OR "dietary supplements" ) AND ( "clinical trial" OR "experimental study" OR randomized OR randomly OR "controlled trial" OR placebo) ) | 3 |
| Open Grey | ("cerebral palsy" OR "non progressive encephalopathy") AND (nutrition* OR diet*) | 1 |
| **TOTAL** | | **25** |

* Search date: June 17th, 2021
