# Supplementary material for: Dietary and nutritional interventions in children with cerebral palsy: A systematic literature review
Source: PLoS One. 2022 Jul 22;17(7):e0271993. doi: 10.1371/journal.pone.0271993 (PMC9307182; doi:10.1371/journal.pone.0271993)
Supplement: S4 Appendix — (DOCX) [file pone.0271993.s006.docx]

**S4 Appendix. Summary of main quantitative results for each study**

**Studies evaluating as main outcome gastric emptying rate, gastroesophageal reflux and related symptoms**

| **Study** | **Interventions** | **Duration of treatment** | **Outcome**  **studied** | **Summary of key results** | **Statistics** | **Main quantitative results (Outcomes not cited did not present significant results)** |
| --- | --- | --- | --- | --- | --- | --- |
| Fried, 1992 | Enteral formulas (A) Whey predominant [60% whey + 40% casein], (B) 100% Whey hydrolysate, (C) 100% Whey hydrolysate with 70% of the fat as MCT | 2 hours for gastric emptying and 1 month for vomiting | Gastric emptying, episodes of vomiting | Whey-based formulas led to a faster gastric emptying and reduced the number of episodes of vomiting. The faster gastric empty rate was obtained with formula B. | Analysis of variance and Student’s t test  (Mean ± SD) | Gastric emptying at 60 minutes  A: 48% ± 19^#^  B: 56% ± 23^#^  C: 59% ± 19^#^  D: 85% ± 11*  Gastric emptying at 120 minutes  A: 24% ± 18^#^  B: 31% ± 26^#^  C: 27% ± 13^#^  D: 69% ± 14*  Episodes of vomiting  A, B and C: 2 ± 2 episodes, range up to 7 episodes  D: 12 ± 11 episodes, range 4 to 35 episodes |
|  | (D) Enteral formula casein predominant (80% casein, 20% soy) |  |  |  |  |  |
| Khoshoo, 1996 | Whey-based enteral isosmolar formula (4g protein, 100% whey) | 48 hours | GER | The whey-based diet significantly reduced the frequency and duration of GER episodes when compared to the casein-based diet. | Student's t test (Mean ± SD) | Number of episodes of GER (p < 0.05)  Whey group: 51 ± 15  Casein group: 73.1 ± 22.8  Duration of GER (p < 0.05)  Whey group: 10.8 ± 5.9  Casein group: 15.2 ± 2.9 |
|  | Casein-based isocaloric and isosmolar enteral formula (3.7g protein, 84% casein, 16% soy) |  |  |  |  |  |
| Graham-Parker, 2001 | Enteral formulas (A) 18% whey; (B) 50% whey | 4 weeks | Emesis, gagging/retching, stool frequency and consistency, volume intakes, degree of irritability and anthropometric parameters. | Diet B caused less gagging/retching than Diet A. There was a slight decrease in stool frequency during the Diet B period as compared to the Diet A period. Stool consistency remained the same with Diet B and became softer than usual during Diet A period | Compared means between groups – did not specify the test  (Mean ± SD) | Gagging/retching  A: 3.8 ± 6.1 vs. B: 2.5± 4.7; p<0.001.  Stool frequency  A: 1.1 ± 1.1; vs. B: 0.8 ± 0.8; p<0.001  Consistency of the stool remained the same with Diet B and became softer than usual during Diet A period (p<0.001). |
|  | The formulas were compared with each other |  |  |  |  |  |
| Savage, 2012 | Enteral formula 50% whey/ 50% casein; or 100% enteral formula partially hydrolyzed whey | 1 week | GER, gastric emptying and symptoms of food intolerance (choking, regurgitation, irritability, regurgitation and pain) | Whey-based formulas led to faster gastric emptying, compared to casein-based formula. Reflux parameters remained unchanged. GI symptoms were less frequent in children who received the formula 50% whey, compared to those who received 100% whey, whose pain scores worsened. | Wilcoxon signed-ranks test, Mann-Whitney rank sum test and Spearman rank correlation  (Median (IQR)) | Total reflux episodes  50% whey: 32 (17.5-19.4) vs. Casein: 55 (24.5-194.5)  100% whey: 25.5 (14.3-68.3) vs. Casein: 41.5 (9-87.5)  Gastric emptying  50% whey: 33.1 (27.5-187.1) vs. Casein: 56.6 (56-394.9)  100% whey: 39 (22.5-90.6) vs. Casein: 59.5 (24-86) |
|  | Standard casein-based enteral formula (82% casein and 18% serum) |  |  |  |  |  |
| Brun, 2012 | Enteral formulas (A) casein 100%; (B) Hydrolyzed whey; (C) Amino acids; or (D) 40% casein/ 60% whey | 4 days | Gastric emptying rate; Postprandial GI symptoms | The shortest gastric emptying time was obtained when the children received the formula D: 40% casein/ 60% serum. For formula D, emptying was significantly faster in children with postprandial symptoms, compared to those without symptoms. | Wilcoxon test, Mann Whitney  test, Fisher exact test, and Friedman test  (Median (IQR)) | Half time of gastric emptying  A: 153.9 (76.4-230.6) vs. B: 82.0 (27.1-169.9) vs. C: 74.4 (34.3-107.3) vs. D: 63.3 (41.9e99.5) (p=0.02)  Gastric emptying coefficient  A: 1.9 (1.8-2.1) vs. B: 2.4 (2.2-2.6) vs. C: 2.5 (2.3-2.7) vs. D: 2.4 (1.9-2.7) (p=0.003)  Time until maximum excretion of 13C  A: 135 (30-195) vs. B: 60 (45-135) vs. C: 60 (45-120) vs. D: 60 (30-105) (p=0.04) |
|  | The formulas were compared with each other |  |  |  |  |  |
| Miyazawa, 2008 | Enteral formula rich in pectin [liquid pectin = 2: 1 (v / v)] | 4 weeks | GER (esophageal pH) and symptoms of GER disease (vomiting, residual gastric volume, wheezing and cough episodes, use of oxygen for dyspnea) | The diet rich in pectin significantly reduced the GER index, the number of GER episodes per day, the duration of the GER and the number of vomit episodes, compared with the diet without pectin. Both the diet rich in pectin and low pectin reduced the cough score, compared to the diet without pectin. | χ2 test, unpaired Student's t test, or Wilcoxon's  signed rank test  (Median (IQR)) | % time pH < 4 at the lower esophagus  No pectin: 9.2% (6.2–22.6) vs. High pectin: 5.0% (3.1–13.1); p < 0.01  % time pH < 4 at the lower esophagus  No pectin: 3.8% (2.9–11.2) vs. High pectin: 1.6%  (0.9–8.9); p < 0.01  Episodes of vomiting  No pectin: 2.5/week (1.0–5.0) vs. High pectin: 1.0 (1.0–1.5), P < 0.05  Cough-score variation (baseline vs. endpoint):  Hight pectin: 8.5/week (1.0–11.5) vs. 2.0/week (0.0–3.0); p < 0.05  Low pectin: 7.0/week (1.0–14.5) vs. 1.0/w (0.0–5.0); p < 0.05 |
|  | Enteral formula with low [liquid pectin = 3: 1 (v / v)] or no pectin |  |  |  |  |  |

Note: *and #: different symbols represent significant differences (p<0.05). GER, Gastroesophageal reflux; GI, gastrointestinal; IQR, Interquartile range; MCT, medium-chain triglycerides; SD, Standard deviation.

**Studies evaluating as main outcome plasma concentration of 25-hydroxyvitamin D**

| **Study** | **Interventions** | **Duration of treatment** | **Outcome**  **studied** | **Summary of key results** | **Statistics** | **Main quantitative results**  **(Outcomes not cited did not present significant results)** |
| --- | --- | --- | --- | --- | --- | --- |
| Le Roy, 2015 | Single dose of 100,000 IU D3 orally | Single dose | Plasma concentrations of 25OHD | Baseline 25OHD was insufficient in 4/10 of placebo group and 1/8 in the intervention group. After 8 weeks, it remained insufficient in 4/10 in the placebo group and reached the desired levels in all participants of the intervention group. The intervention was not associated with any AE. | Fisher exact test | 25OHD status:  Placebo: insufficient in 4/10  VitD3: insufficient in 0/8 |
|  | Placebo |  |  |  |  |  |
| Kilpinen-Loisa, 2007 | 1000 IU of vitamin D3 orally 5 days/week for 10 weeks | 10 weeks | Plasma concentrations of 25OHD, Calcium homeostasis parameters and bone metabolism | The plasma concentration of 25OHD increased significantly in the supplemented group and decreased in the control group. The intervention was not associated with hypercalcemia or other AE. | Mann-Whitney U test  Medians (range) | 25OHD (nmol/ L)  Control: 37 (24 – 74)  VitD3: 56 (39 – 88); p < 0.0001 |
|  | Observational (without placebo) |  |  |  |  |  |

Note: 25OHD, 25-hydroxyvitamin D; AE, Adverse effects; ITV, Intervention.

**Studies evaluating as main outcome anthropometric measurements and nutritional status**

| **Study** | **Interventions** | **Duration of treatment** | **Outcome**  **studied** | **Summary of key results** | **Statistics** | **Main quantitative results**  **(Outcomes not cited did not present significant results)** |
| --- | --- | --- | --- | --- | --- | --- |
| Sevilla Paz Soldán, 2018 | 13 vitamins and 6 minerals according to age + 10 mL of lipid mixture containing: Coconut oil (35%), olive oil (35%), marine fish oil (15%), soybean oil (15%). | 6 months | Weight gain and body composition, disability, lipid profile and psychomotor development | The group that received the lipid mixture presented better psychomotor development scores, anthropometric indicators and lipid profile at the end of follow-up | Wilcoxon's  signed rank test  (Mean ± SD) | Not clear. |
|  | 13 vitamins and 6 minerals according to age |  |  |  |  |  |
| Patrick, 1986 | Intensive nasogastric tube-feeding | 5 weeks | Weight gain, triceps skinfold and arm muscle circumference | Intervention increased 10 to 46% of body weight in 4 to 5 weeks with approximately 50% increase in energy intake. The gain in arm muscle circumference and triceps skinfold after the intervention suggests that both lean tissues and fat increased. | Student’s t test  (Mean) | Wheigh change (%)  Treatment: 33.1 vs. Control: -0.6; p<0.01  Triceps skinfold  Before feeding: 7.0 ± 0.94  After feeding: 12.8 ± 1.5  Mid-arm muscle circumference  Before feeding: 13.1 ± 0.35  After feeding: 14.9 ± 1.0 |
|  | Best oral feeding that could be achieved |  |  |  |  |  |

Note: ITV, Intervention; SD, Standard deviation.

**Studies evaluating as main outcome constipation and fecal characteristics.**

| **Study** | **Interventions** | **Duration of treatment** | **Outcome**  **studied** | **Summary of key results** | **Statistics** | **Main quantitative results**  **(Outcomes not cited did not present significant results)** |
| --- | --- | --- | --- | --- | --- | --- |
| García-Contreras, 2020 | 1 × 10e8 cfu of L. reuteri DSM 17938 and 4 g maltodextrin | 28 days | Fecal characteristics (pH, consistency, frequency and microbiota) | Intervention with L. reuteri DSM 17938 and/or inulin agave significantly improved stool characteristics. | Wilcoxon, Cochran’s Q test, and McNemar test, Mann–Whitney U test, Friedman tests  (frequencies and median (IQR)) | Stool pH significantly decreased (p=0.014) and stool frequency/week increased (p=0.034) after intervention, only in Probiotic group (median (IQR) values not informed)  Frequency of hard stool compared to placebo  Probiotic: basal 90% vs. final 60%; p=0.121  Synbiotic: basal 70% vs. final 40%; p=0.063  Prebiotic: basal 80% vs. final 30%; p=0.031  Plabebo: basal 43% vs. final 43%  Gut microbiota  L. reuteri: Probiotic (5.97 ± 1.7 log10 cells/g) vs.Placebo (4.2 ± 1.9 log10 cells/g); p = 0.001 |
|  | 1 × 10e8 cfu of L. reuteri DSM 17938 and 4 g inulin agave |  |  |  |  |  |
|  | 4 g of agave inulin and 5 drops of an oil mixture containing both medium-chain triglycerides and sunflower oil |  |  |  |  |  |
|  | 4 g maltodextrin and 5 drops of oil mixture |  |  |  |  |  |
| Hassanein, 2021 | Oral magnesium sulfate solution 4% (4 mg elemental magnesium/mL, 1 mL/kg/day), respecting daily limit (65, 110 and 350 mg/day for 1-3 years, 3-8 years and above 9 years, respectively). | 1 month | Fecal characteristics (consistency, frequency and daily time for evacuation) | The use of oral magnesium sulfate resulted in a significant improvement in constipation scores, consistency and fecal frequency after 1 month, when compared to placebo. | Two-sample t test, paired t test, chi-squared test, multinomial logistic  Regression. | Stool frequency ≥ 3/week (%)  O-Mg: 75.6 vs. Placebo 21.4; p<0.001  Bristol stool scale ≥ 3 (%)  O-Mg: 91.1 vs. Placebo 33.4; p<0.001  Bowel movements/week (mean ± SD)  O-Mg: 4.04 ± 1.69 vs. Placebo 2.29 ± 0.67; p<0.001  Bowel evacuation > 2h/day (%)  O-Mg: 22.0 vs. Placebo 42.9; p<0.001 |
|  | Placebo (Saline Solution) |  |  |  |  |  |

Note: IQR, Interquartile range.

| **Study** | **Groups (n)** | **Interventions** | **Duration of treatment** | **Outcome**  **studied** | **Summary of key results** | **Statistics** | **Main quantitative results**  **(Outcomes not cited did not present significant results)** |
| --- | --- | --- | --- | --- | --- | --- | --- |
| Leal-Martínez, 2020 | IG (10) | Nutritional support system (shake-based diet with functional ingredients, high levels of vegetables, fruits, cereals, roots and fish and supplementation with glutamine, arginine, folic acid, nicotinic acid, zinc, selenium, cholecalciferol, ascorbic acid, spirulina, vegetable protein, PUFAs n-3 and probiotics.) | 13 weeks | Gross-motor function | Children with nutritional support showed superior motor function after 7 and 13 weeks after the beginning of the intervention, especially the parameters "standing up" and "walking". | Mann Whitney U test, Kruskal Wallis followed by the Dunn posthoc test,.Rosenthal’s r  (Mean ± SD) | Crawling at 13^th^ week  FG: 57.72 ± 17; CG: 54.38 ± 30; IG: 69.86 ± 26; p = 0.03  Standing at 7^th^ week  IG: 26.19 ± 8.2 vs. FG: 10.1 ± 4.9; p = 0.0004, and vs. CG 12.98 ± 7.2; p = 0.003  Standing at 13^th^ week  IG: 30.9 ± 8.7 vs. FG: 13.32 ± 7.4; p = 0.0002, and vs. CG 17.29 ± 8.1; p = 0.003  Walking at 7^th^ week  IG: 24.65 ± 6.1 vs. FG: 18.47 ± 5.7; p = 0.01, and vs. CG 19.45 ± 5.6; p = 0.03  Walking at 13^th^ week  IG: 34.68 ± 7.3 vs. FG: 19.86 ± 6.0; p = 0.0003, and vs. CG 22.51 ± 5.9; p = 0.001 |
|  | CG (10) | Diet recommended by WHO) |  |  |  |  |  |
|  | FG (10) | Monitoring of the usual diet |  |  |  |  |  |
| Omar, 2021 | ITV (34) | Lactoferrin (oral, 30% bovine iron), 100 mg/day | 4 weeks | Hemoglobin variation, serum iron, biochemical parameters, adherence to therapy, AE | All laboratory markers of anemia were improved with the use of lactoferrin and the polymised ferric hydroxide complex. The variation of hemoglobin and ferritin was significantly higher in children who received lactoferrin, who additionally had a lower incidence of constipation as an AE | Student’s t-test, Mann-Whitney test  Mean (95%CI) | Hemoglobin adjusted change (g/dL)  IPC: 0.75 (0.49–0.99) vs. Lf : 1.36 (1.12–1.60); p=0.001  Serum ferritin adjusted change (ng/mL)  IPC: 2.88 (0.97–4.79) vs. Lf : 5.79 (3.94–7.65); p=0.033  Constipation (%)  IPC: 12.5 vs. Lf : 0.0; p=0.049 |
|  | Control (32) | Iron hydroxide polymaltose complex (oral, 6 mg/kg/day elemental iron divided into 2 doses) |  |  |  |  |  |
| Mlinda, 2018 | ITV (69) | Group and individual nutritional education, caregivers' training on positioning during feeding and occupational therapy for oral motor and functional skills. | 12 months | Feeding skills and caregiver-child interaction during feeding | Caregivers in the intervention group reported significant improvements in positioning skills, feeding speed, child involvement during feeding and child-caregiver interaction, in addition to lower stress and improvement of the child's mood during feeding | Logistic regression analysis adjusted for residual confounding  from demographic characteristics | More caregivers appropriately positioned the children (AOR = 5.29; 95% CI: 2.00–13.96), fed children slowly (AOR: 5.17, 95% CI: .99–13.44), and involved the child during the feeding process (AOR = 3.46; 95% CI: 1.42–8.44). During feeding, caregiver's reported being less stressed (AOR = 2.53, 95% CI: 1.04–6.13) and the child's mood was more likely to be reported as improved (AOR = 3.15, 95% CI: 1.33–7.474). |
|  | Control (49) | Routine general care offered in clinics regularly |  |  |  |  |  |

**Studies evaluating others main outcomes as follows: motor function, laboratory markers of anemia and feeding skills**

Note: AE, Adverse effects; Interquartile range; ITV, Intervention.
