# Supplementary material for: Dietary and nutritional interventions in children with cerebral palsy: A systematic literature review
Source: PLoS One. 2022 Jul 22;17(7):e0271993. doi: 10.1371/journal.pone.0271993 (PMC9307182; doi:10.1371/journal.pone.0271993)
Supplement: Attachment — Submitted filename: PONE-D-21-30189_Reviewed manuscript.pdf [file pone.0271993.s007.pdf]

PLOS ONE

Dietary and nutritional interventions in children with cerebral palsy: a systematic literature review

--Manuscript Draft--

**Manuscript Number:**	PONE-D-21-30189
**Article Type:**	Research Article
**Full Title:**	Dietary and nutritional interventions in children with cerebral palsy: a systematic literature review
**Short Title:**	Dietary and nutritional interventions in children with cerebral palsy
**Corresponding Author:**	Fernanda Rebelo<br>Oswaldo Cruz Foundation: Fundacao Oswaldo Cruz<br>BRAZIL
**Keywords:**	Cerebral palsy; nutritional interventions; dietary interventions; children; systematic literature review
**Abstract:**	Background: Cerebral palsy is an extremely severe brain injury associated with multiple nutritional and clinical issues, such as underweight, gastroesophageal reflux, constipation, and nutrient deficiency. Evidence-based dietary and nutritional interventions may improve the quality of life of children with cerebral palsy. Aim: Systematically review randomized clinical trials evaluating nutritional and dietary interventions in the clinical, nutritional, and neurodevelopmental aspects of children with cerebral palsy. Methods: A search was performed in electronic databases (LILACS, Medline, Web of Science, Embase, Scopus, Cochrane Library, ClinicalTrials.gov, Brazilian Digital Library of Theses and Dissertations, ProQuest Dissertations and Theses Database, OpenGrey) using keywords such as: ("cerebral palsy" OR “chronic non progressive encephalopathy”) AND (child OR children) AND (nutrients OR nutrition OR diet). Eligible studies must be randomized clinical trials, include children between 2 and 12 years old, and evaluate the effect of nutritional or dietetic interventions on clinical, nutritional or neurodevelopmental outcomes. Risk of bias was investigated using the RoB-2 tool. The study was registered on PROSPERO (CRD42020181284) Results: Fifteen studies were selected. Positive results included the use of whey-based or pectin-enriched enteral formulas for gastroesophageal reflux (n=6); 25-hydroxy-vitamin D supplementation for hypovitaminosis D (n=2); supplementation with lipid mixture or diet with high-density energy for improvements in anthropometric measures (n=2); supplementation with probiotics, prebiotics, symbiotics or magnesium for constipation (n=2); nutritional support system for gross motor function (n=1); lactoferrin and iron hydroxide polymaltose for iron deficiency anemia (n=1); and educational intervention to improve feeding skills (n=1). The overall risk of bias was high for 60% of the studies, and some concerns were raised for the remaining 30%. Conclusion: Some promising dietary and nutritional interventions may promote important clinical improvements for patients with cerebral palsy. However, evidence is weak, as few clinical trials have been published with many methodological errors, leading to a high risk of bias.
**Order of Authors:**	Fernanda Rebelo
	Isabela Rodrigues Mansur
	Teresa Cristina Miglioli
	Maria Dalva Baker Meio
	Saint Clair Gomes Gomes Junior
**Additional Information:**	
**Question**	**Response**
**Financial Disclosure**<br><br>Enter a financial disclosure statement that	This study was funded by the National Research Council (CNPq, process number 442551/2019-3). The funders had no role in the study design, data collection and analysis, decision to publish, or preparation of the manuscript.

describes the sources of funding for the work included in this submission. Review the submission guidelines for detailed requirements. View published research articles from *PLOS ONE* for specific examples.<br><br>This statement is required for submission and **will appear in the published article** if the submission is accepted. Please make sure it is accurate.<br><br>**Unfunded studies**<br>Enter: *The author(s) received no specific funding for this work.*<br><br>**Funded studies**<br>Enter a statement with the following details:<br>• Initials of the authors who received each award<br>• Grant numbers awarded to each author<br>• The full name of each funder<br>• URL of each funder website<br>• Did the sponsors or funders play any role in the study design, data collection and analysis, decision to publish, or preparation of the manuscript?<br>• **NO** - Include this sentence at the end of your statement: *The funders had no role in study design, data collection and analysis, decision to publish, or preparation of the manuscript.*<br>• **YES** - Specify the role(s) played.<br><br>* typeset	
**Competing Interests**<br><br>Use the instructions below to enter a competing interest statement for this submission. On behalf of all authors, disclose any competing interests that could be perceived to bias this work—acknowledging all financial support and any other relevant financial or non-financial competing interests.<br><br>This statement is required for submission and **will appear in the published article** if the submission is accepted. Please make sure it is accurate and that any funding	The authors have declared that no competing interests exist.

sources listed in your Funding Information later in the submission form are also declared in your Financial Disclosure statement.<br><br>View published research articles from *PLOS ONE* for specific examples.<br><br>**NO authors have competing interests**<br><br>Enter: *The authors have declared that no competing interests exist.*<br><br>**Authors with competing interests**<br><br>Enter competing interest details beginning with this statement:<br><br>*I have read the journal's policy and the authors of this manuscript have the following competing interests: [insert competing interests here]*<br><br>* typeset	
**Ethics Statement**<br><br>Enter an ethics statement for this submission. This statement is required if the study involved:<br><br>• Human participants<br>• Human specimens or tissue<br>• Vertebrate animals or cephalopods<br>• Vertebrate embryos or tissues<br>• Field research<br><br>Write "N/A" if the submission does not require an ethics statement.<br><br>General guidance is provided below. Consult the submission guidelines for detailed instructions. **Make sure that all information entered here is included in the Methods section of the manuscript.**	N/A

**Format for specific study types**<br><br>**Human Subject Research (involving human participants and/or tissue)**<br>• Give the name of the institutional review board or ethics committee that approved the study<br>• Include the approval number and/or a statement indicating approval of this research<br>• Indicate the form of consent obtained (written/oral) or the reason that consent was not obtained (e.g. the data were analyzed anonymously)<br><br>**Animal Research (involving vertebrate animals, embryos or tissues)**<br>• Provide the name of the Institutional Animal Care and Use Committee (IACUC) or other relevant ethics board that reviewed the study protocol, and indicate whether they approved this research or granted a formal waiver of ethical approval<br>• Include an approval number if one was obtained<br>• If the study involved *non-human primates*, add *additional details* about animal welfare and steps taken to ameliorate suffering<br>• If anesthesia, euthanasia, or any kind of animal sacrifice is part of the study, include briefly which substances and/or methods were applied<br><br>**Field Research**<br><br>Include the following details if this study involves the collection of plant, animal, or other materials from a natural setting:<br>• Field permit number<br>• Name of the institution or relevant body that granted permission	
**Data Availability**<br><br>Authors are required to make all data underlying the findings described fully available, without restriction, and from the time of publication. PLOS allows rare exceptions to address legal and ethical concerns. See the PLOS Data Policy and FAQ for detailed information.	Yes - all data are fully available without restriction

A Data Availability Statement describing where the data can be found is required at submission. Your answers to this question constitute the Data Availability Statement and **will be published in the article**, if accepted.<br><br>**Important:** Stating 'data available on request from the author' is not sufficient. If your data are only available upon request, select 'No' for the first question and explain your exceptional situation in the text box.<br><br>Do the authors confirm that all data underlying the findings described in their manuscript are fully available without restriction?	
**Describe where the data may be found in full sentences. If you are copying our sample text, replace any instances of XXX with the appropriate details.**<br><br>• If the data are **held or will be held in a public repository**, include URLs, accession numbers or DOIs. If this information will only be available after acceptance, indicate this by ticking the box below. For example: *All XXX files are available from the XXX database (accession number(s) XXX, XXX.*).<br>• If the data are all contained **within the manuscript and/or Supporting Information files**, enter the following: *All relevant data are within the manuscript and its Supporting Information files.*<br>• If neither of these applies but you are able to provide **details of access elsewhere**, with or without limitations, please do so. For example:<br><br>*Data cannot be shared publicly because of [XXX]. Data are available from the XXX Institutional Data Access / Ethics Committee (contact via XXX) for researchers who meet the criteria for access to confidential data.*<br><br>*The data underlying the results presented in the study are available from (include the name of the third party*	All relevant data are within the manuscript and its Supporting Information files.

and contact information or URL).<br>• This text is appropriate if the data are owned by a third party and authors do not have permission to share the data.<br><br>* typeset	
Additional data availability information:	

Powered by Editorial Manager® and ProduXion Manager® from Aries Systems Corporation

Manuscript Click here to access/download;Manuscript;Manuscript.docx

Dietary and nutritional interventions in children with cerebral palsy: a systematic literature review

Fernanda Rebelo[1*]
Isabela Rodrigues Mansur[1,2]
Teresa Cristina Miglioli[1,#a]
Maria Dalva Baker Meio[1]
Saint Clair Gomes Junior[1]

[1]Clinical Research Unit, National Institute of Women, Children and Adolescents Health Fernandes Figueira (IFF), Oswaldo Cruz Foundation (Fiocuz), Rio de Janeiro, RJ, Brazil.
[2]Undergraduate Program in Nutrition, Fluminense Federal University (UFF), Niterói, RJ, Brazil
[#a]Nutrition Department, Iguaçu University (UNIG), Nova Iguaçu, RJ, Brazil

*Corresponding author
e-mail: frebelos@gmail.com

Abstract

Background: Cerebral palsy is an extremely severe brain injury associated with multiple nutritional and clinical issues, such as underweight, gastroesophageal reflux, constipation, and nutrient deficiency. Evidence-based dietary and nutritional interventions may improve the quality of life of children with cerebral palsy. **Aim:** Systematically review randomized clinical trials evaluating nutritional and dietary interventions in the clinical, nutritional, and neurodevelopmental aspects of children with cerebral palsy. **Methods:** A search was performed in electronic databases (LILACS, Medline, Web of Science, Embase, Scopus, Cochrane Library, ClinicalTrials.gov, Brazilian Digital Library of Theses and Dissertations, ProQuest Dissertations and Theses Database, OpenGrey) us[illegible] keywords such as: ("cerebral palsy" OR “chronic non progressive encephalopathy”) AND (child OR children) AND (nutrients OR nutrition OR diet). Eligible studies ~~must be~~ randomized clinical trials, ~~include~~ children between 2 and 12 years old, and ~~evaluate~~ the effect of nutritional or dietetic interventions on clinical, nutritional or neurodevelopmental outcomes. Risk of bias was investigated using the RoB-2 tool. The study was registered on PROSPERO (CRD42020181284) **Results:** Fifteen studies were selected. Positive results included the use of whey-based or pectin-enriched enteral formulas for gastroesophageal reflux (n=6); 25-hydroxy-vitamin D supplementation for hypovitaminosis D (n=2); supplementation with lipid mixture or diet with high-density energy for improvements in anthropometric measures (n=2); supplementation with probiotics, prebiotics, symbiotics or magnesium for constipation (n=2); nutritional support system for gross motor function (n=1); lactoferrin and iron hydroxide polymaltose for iron deficiency anemia (n=1); and educational intervention to improve feeding skills (n=1). The overall risk of bias was high for 60% of the studies, and some concerns were raised for the remaining 30%. **Conclusion:** Some promising dietary and nutritional interventions may promote important clinical improvements for patients with cerebral palsy. However, evidence is weak, as few clinical trials have been published with many methodological errors, leading to a high risk of bias.

Keywords: Cerebral palsy, nutritional interventions, dietary interventions, children, systematic review.

Introduction

Cerebral palsy (also known as chronic non-progressive encephalopathy) is an extremely severe, disabling brain injury with repercussions that are not limited to the individual, affecting their families and society. This health condition comprises a group of permanent and non-progressive disorders of movement and posture secondary to injury, dysfunction, or damage to fetal or infant brain development. These motor disorders are often accompanied by changes in sensory, perception, cognition, communication, behavior, epileptic seizures, secondary musculoskeletal problems, and important comorbidities [1,2]. These comorbidities include nutritional issues, such as underweight, gastroesophageal reflux, constipation, and deficiency of micro- and macronutrients [3–6].

The influence of nutrition on changes in the functioning of the human body is recognized, both in the causal chain of diseases and in the modification of responses in already established diseases. Specific nutrients can act in enzymatic processes, such as zinc and magnesium, or in the formation of hemoglobin, such as iron, in bone formation, such as vitamin D and calcium, in the formation of muscle mass, such as proteins [7–9]. The use of alternative enteral formulas or addiction of fibers, probiotics, prebiotics, lipids or fatty acids in the diet can act as an adjunct in the treatment of gastroesophageal reflux, constipation, undernutrition and others [10–12]. Therefore, nutritional interventions can act in several ways to improve the quality of life of patients with cerebral palsy.

The identification of dietary or nutritional interventions with good results for the treatment of common disorders in children with cerebral palsy, with high-level scientific evidence, is important for the construction of protocols and for the guidance of evidence-based practice. It provides subsidies for the management and organization of nutritional care in public health, promoting the reduction of morbidity and hospital costs and the improvement of the quality of life of children with cerebral palsy. Thus, the present systematic review aims to synthetize the results of randomized clinical trials evaluating the effect of nutritional and dietary interventions in clinical, nutritional and neurodevelopmental outcomes of children with cerebral palsy.

Methods

This is a systematic literature review (SLR) conducted following the recommendations of the Cochrane handbook for systematic review of interventions and reported according to the Preferred Reporting Items for Systematic reviews and Meta-Analyses (PRISMA) Statement [13,14]. This SLR was registered on PROSPERO under the number CRD42020181284. Our aim is to determine the effect of nutritional and dietary interventions on clinical, nutritional and neurodevelopmental outcomes in children with cerebral palsy.

Search strategy

We performed an automatic literature search in the following electronic databases: LILACS, Medline, Web of Science, Embase, Scopus, Cochrane Library, ClinicalTrials.gov, Brazilian Digital Library of Theses and Dissertations, ProQuest Dissertations and Theses Database, and OpenGrey. The following keywords were used and adapted for each database: ("cerebral palsy" OR “chronic non progressive encephalopathy”) AND (child OR children OR childhood) AND (nutrients OR nutrition OR diet). Moreover, sensitive strategies were applied to identify randomized controlled trials. A search summary with a complete strategy for each database can be found in **S1 Appendix**. Finally, we performed a backward search, manually checking the reference lists of the eligible studies. Our literature search was first performed in May 2020 and updated on June 18th, 2021.

To be included in the SLR, the studies had to be randomized clinical trials, include children between 2 and 12 years old, and evaluate the effect of nutritional or dietetic interventions on clinical, nutritional, neurodevelopmental and/or seizure control outcomes. There were no restrictions regarding year, idiom or type of publication.

Studies selection

The retrieved references were managed using Covidence software [15]. First, titles and abstracts were screened. Second, the full texts were checked for eligibility. Both steps were performed by two independent reviewers (IRM, TCM). Disagreements were resolved by a third investigator (FR). When multiple reports of the same study/database were identified, the more detailed report was selected for the SLR. When the reference was a register (in ClinicalTrials.gov or Cochrane Library) with no publications associated, we searched for the principal investigator productions to obtain the results.

Data extraction

Data extraction was performed using a structured questionnaire by two authors (IRM, TCM). A third author (FR) reviewed the data and resolved any disagreements. The following information was extracted from the selected studies: year of publication, country and setting where the study was conducted, follow-up period, sample size, recruitment and randomization process, losses of follow-up, inclusion and exclusion criteria, sample characteristics (age, gender, functional mobility), intervention tested, control group, blindness, time from intervention to outcome evaluation, methods for outcome evaluation, statistical analysis and summary of the main results.

Risk-of-bias assessment

The risk-of-bias assessment was performed using the revised Cochrane tool for assessing risk of bias in randomized trials (RoB 2 tool) [16] by two independent investigators (IRM, TCM). Differences were solved by a third reviewer (FR).

A summary risk of bias table was generated using the Risk- of- bias VISualization (robvis) tool [17]. The complete RoB 2 evaluation is presented as supplemental material (**S1 Table)**. Studies

were classified with an overall high risk of bias when at least one domain was judged as having a high risk of bias or when some concerns were found in at least three domains.

Results

A total of 508 references were obtained from electronic databases. After exclusion of duplicates, 399 references were screened against titles and abstracts, and 43 full-text studies were assessed for eligibility. Fifteen studies met all eligibility criteria and were selected for the SLR. A flowchart with a detailed selection process is described in **Fig 1**. The references excluded after full-text evaluation and the respective reasons are listed in **S2 Table**.

Figure 1. Flow-chart illustrating the search and selection process.

We observed a preponderance of studies performed in North America (3 in Canada, 2 in Mexico, 1 in the United States), followed by Africa (2 in Egypt, 1 in Tanzania), South America (1 in Bolivia, 1 in Chile), Europe (1 in Finland), Asia (1 in Japan) and Oceania (1 in Australia). The first record is the study from Patrick and colleagues from ~~1986 in Canada~~ [18]. In the following twenty years publications were scarce, with only 4 studies. In 2007 publications became more frequent, with 11 studies in the last 14 years (**Tables 1-5**).

Considering the main outcomes investigated, studies were classified into 5 groups: (1) gastric emptying rate, gastroesophageal reflux (GER) and related symptoms; (2) plasma concentration of 25-hydroxyvitamin D; (3) anthropometric measures and nutritional status; (4) constipation and stool characteristics; and (5) other outcomes evaluated by only one study (**Tables 1-5**).

Table 1. Characteristics of the studies evaluating as main outcome gastric emptying rate, gastroesophageal reflux and related symptoms.

Study	Country	N	Age (Years)	Sex		Functional mobility	Groups (n)	Interventions	Duration of treatment	Outcome studied	Summary of key results
				F (%)	M (%)						
Fried, 1992	Canada	9*	3 - 18	NA	NA	Spastic quadriplegia	ITV (9)	Enteral formulas (A) Whey predominant [60% whey + 40% casein], (B) 100% Whey hydrolysate, (C) 100% Whey hydrolysate with 70% of the fat as MCT	2 hours for gastric emptying and 1 month for vomiting	Gastric emptying, episodes of vomiting	Whey-based formulas led to a faster gastric emptying and reduced the number of episodes of vomiting. The faster gastric empty rate was obtained with formula B.
							Control (9)	Enteral formula casein predominant (80% casein, 20% soy)			
Khoshoo, 1996	USA	10*	4.5 - 14.5	40	60	NA	ITV (10)	Whey-based enteral isosmolar formula (4g protein, 100% whey)	48 hours	GER	The whey-based diet significantly reduced the frequency and duration of GER episodes when compared to the casein-based diet.
							Control (10)	Casein-based isocaloric and isosmolar enteral formula (3.7g protein, 84% casein, 16% soy)			
Graham-Parker, 2001	Canada	20*	2 - 9	NA	NA	NA	ITV (20)	Enteral formulas (A) 18% whey; (B) 50% whey	4 weeks	Emesis, gagging/retching, stool frequency and consistency, volume intakes, degree of irritability and anthropometric parameters.	Diet B caused less gagging/retching than Diet A. There was a slight decrease in stool frequency during the Diet B period as compared to the Diet A period. Stool consistency remained the same with Diet B and became softer than usual during Diet A period
							Control (20)	The formulas were compared with each other			
Savage, 2012	Australia	13*	2.4 - 15.4	38.5	61.5	Spastic quadriplegia: 53.8%<br>Spastic Diplegia: 23.1%<br>Hypotonia: 15.4%<br>Dyskinetics: 7.7%	ITV (13)	Enteral formula 50% whey/ 50% casein; or 100% enteral formula partially hydrolyzed whey	1 week	GER, gastric emptying and symptoms of food intolerance (choking, regurgitation, irritability, regurgitation and pain)	Whey-based formulas led to faster gastric emptying, compared to casein-based formula. Reflux parameters remained unchanged. GI symptoms were less frequent in children who received the formula 50% whey, compared to those who received 100% whey, whose pain scores worsened.
							Control (13)	Standard casein-based enteral formula (82% casein and 18% serum)			
Brun, 2012	Norway	15*	4 - 15	46.7	53.3	NA	ITV (15)	Enteral formulas (A) casein 100%; (B) Hydrolyzed whey; (C) Amino acids; or (D) 40% casein/ 60% whey	4 days	Gastric emptying rate; Postprandial GI symptoms	The shortest gastric emptying time was obtained when the children received the formula D: 40% casein/ 60% serum. For formula D, emptying was significantly faster in children with postprandial symptoms, compared to those without symptoms.
							Control (15)	The formulas were compared with each other			

Miyazawa, 2008	Japan	18	11.7 ± 4.4	11.1	88.9	NA	ITV 1 (9)	Enteral formula rich in pectin [liquid pectin = 2: 1 (v / v)]	4 weeks	GER (esophageal pH) and symptoms of GER disease (vomiting, residual gastric volume, wheezing and cough episodes, use of oxygen for dyspnea)	The diet rich in pectin significantly reduced the GER index, the number of GER episodes per day, the duration of the GER and the number of vomit episodes, compared with the diet without pectin. Both the diet rich in pectin and low pectin reduced the cough score, compared to the diet without pectin.
							Control (9)	Enteral formula with low [liquid pectin = 3: 1 (v / v)] or no pectin			

Note: * Cross-over study. GER, Gastroesophageal reflux; GI, gastrointestinal; ITV, Intervention; MCT, medium-chain triglycerides.

Table 2. Characteristics of the studies evaluating as main outcome plasma concentration of 25-hydroxyvitamin D

Study	Country	N	Age (Years)	Sex		Functional mobility	Groups (n)	Interventions	Duration of treatment	Outcome studied	Summary of key results
				F (%)	M (%)						
Le Roy, 2015	Chile	18	9.9 (6.2 - 13.5)	36.7	63.3	GMFCS level I 3.3% level II 3.3% level V 93.3%	ITV (8)	Single dose of 100,000 IU D3 orally	Single dose	Plasma concentrations of 25OHD	Baseline 25OHD was insufficient in 4/10 of placebo group and 1/8 in the intervention group. After 8 weeks, it remained insufficient in 4/10 in the placebo group and reached the desired levels in all participants of the intervention group. The intervention was not associated with any AE.
							Control (10)	Placebo			
Kilpinen-Loisa, 2007	Finland	45	9 - 18	43.2	56.8	GMFCS level III 34% level IV 32% level V 34%	ITV (22)	1000 IU of vitamin D3 orally 5 days/week for 10 weeks	10 weeks	Plasma concentrations of 25OHD, Calcium homeostasis parameters and bone metabolism	The plasma concentration of 25OHD increased significantly in the supplemented group and decreased in the control group. The intervention was not associated with hypercalcemia or other AE.
							Control (23)	Observational (without placebo)			

Note: 25OHD, 25-hydroxyvitamin D; AE, Adverse effects; GMFCS, Gross Motor Function Classification System; ITV, Intervention.

Table 3. Characteristics of the studies evaluating as main outcome anthropometric measurements and nutritional status

Study	Country	N	Age (Years)	Sex		Functional mobility	Groups (n)	Interventions	Duration of treatment	Outcome studied	Summary of key results
				F (%)	M (%)						
Sevilla Paz Soldán, 2018	Bolivia	14	< 5	50	50	NA	ITV (7)	13 vitamins and 6 minerals according to age + 10 mL of lipid mixture containing: Coconut oil (35%), olive oil (35%), marine fish oil (15%), soybean oil (15%).	6 months	Weight gain and body composition, disability, lipid profile and psychomotor development	The group that received the lipid mixture presented better psychomotor development scores, anthropometric indicators and lipid profile at the end of follow-up
							Control (7)	13 vitamins and 6 minerals according to age			
Patrick, 1986	Canada	15	2.8 - 15.8	40	60	NA	ITV (10)	Intensive nasogastric tube-feeding	5 weeks	Weight gain, triceps skinfold and arm muscle circumference	Intervention increased 10 to 46% of body weight in 4 to 5 weeks with approximately 50% increase in energy intake. The gain in arm muscle circumference and triceps skinfold after the intervention suggests that both lean tissues and fat increased.
							Control (5)	Best oral feeding that could be achieved			

Note: ITV, Intervention.

Table 4. Characteristics of the studies evaluating as main outcome constipation and fecal characteristics.

Study	Country	N	Age (Years)	Sex		Functional mobility	Groups (n)	Interventions	Duration of treatment	Outcome studied	Summary of key results
				F (%)	M (%)						
García-Contreras, 2020	Mexico	49	1 - 5	38%	62%	Levels IV and V	Probiotic (12)	1 × 10e8 cfu of L. reuteri DSM 17938 and 4 g maltodextrin	28 days	Fecal characteristics (pH, consistency, frequency and microbiota)	Intervention with L. reuteri DSM 17938 and/or inulin agave significantly improved stool characteristics.
							Symbiotic (13)	1 × 10e8 cfu of L. reuteri DSM 17938 and 4 g inulin agave			
							Prebiotic (13)	4 g of agave inulin and 5 drops of an oil mixture containing both medium-chain triglycerides and sunflower oil			
							Placebo (11)	4 g maltodextrin and 5 drops of oil mixture			
Hassanein, 2021	Egypt	87	2 - 12	49%	51%	GMFCS III to V	ITV (45)	Oral magnesium sulfate solution 4% (4 mg elemental magnesium/mL, 1 mL/kg/day), respecting daily limit (65, 110 and 350 mg/day for 1-3 years, 3-8 years and above 9 years, respectively).	1 month	Fecal characteristics (consistency, frequency and daily time for evacuation)	The use of oral magnesium sulfate resulted in a significant improvement in constipation scores, consistency and fecal frequency after 1 month, when compared to placebo.
							Control (42)	Placebo (Saline Solution)			

Note: GMFCS, Gross Motor Function Classification System; ITV, Intervention

Table 5. Characteristics of the studies evaluating others main outcomes as follows: motor function, laboratory markers of anemia and feeding skills

Study	Country	N	Age (Years)	Sex		Functional mobility	Groups (n)	Interventions	Duration of treatment	Outcome studied	Summary of key results
				F (%)	M (%)						
Leal-Martínez, 2020	Mexico	30	4 - 12	40%	60%	GMFCS III	ITV (10)	Nutritional support system (shake-based diet with functional ingredients, high levels of vegetables, fruits, cereals, roots and fish and supplementation with glutamine, arginine, folic acid, nicotinic acid, zinc, selenium, cholecalciferol, ascorbic acid, spirulina, vegetable protein, PUFAs n-3 and probiotics.)	13 weeks	Gross-motor function	Children with nutritional support showed superior motor function after 7 and 13 weeks after the beginning of the intervention, especially the parameters "standing up" and "walking".
							Control (10)	Diet recommended by WHO)			
							Follow-up (10)	Monitoring of the usual diet			
Omar, 2021	Egypt	70	1 - 10	50%	50%	GMFCS level I 7.5% level II 18.2% level III 18.2% level IV 19.7% level V 36.4%	ITV (34)	Lactoferrin (oral, 30% bovine iron), 100 mg/day	4 weeks	Hemoglobin variation, serum iron, biochemical parameters, adherence to therapy, AE	All laboratory markers of anemia were improved with the use of lactoferrin and the polymised ferric hydroxide complex. The variation of hemoglobin and ferritin was significantly higher in children who received lactoferrin, who additionally had a lower incidence of constipation as an AE
							Control (32)	Iron hydroxide polymaltose complex (oral, 6 mg/kg/day elemental iron divided into 2 doses)			
Mlinda, 2018	Tanzania	118	0 - 5	52.7	47.3	Spastic: 56.4% Quadriplegic: 14.5% Hypotonic: 16.4% Mixed: 12.7% Severity Moderate: 48.2% Severe: 51.8%	ITV (69)	Group and individual nutritional education, caregivers' training on positioning during feeding and occupational therapy for oral motor and functional skills.	12 months	Feeding skills and caregiver-child interaction during feeding	Caregivers in the intervention group reported significant improvements in positioning skills, feeding speed, child involvement during feeding and child-caregiver interaction, in addition to lower stress and improvement of the child's mood during feeding
							Control (49)	Routine general care offered in clinics regularly			

Note: AE, Adverse effects; GMFCS, Gross Motor Function Classification System; ITV, Intervention.

The group of studies with main outcomes involving gastric emptying, GER and related symptoms comprised six works [12,19–23]. Five of the six studies [12,19,21–23] were crossover trials that modified the source of protein in the enteral formulas, comparing different proportions of whey and casein. In summary, formulas with higher proportions of whey seem to accelerate gastric emptying, which may contribute to the reduction of GER and diminish symptoms like gagging and retching. However, one study [21] observed higher pain scores in children receiving formula 100% whey than in those receiving formula 50% whey and 50% casein, while another study [12] reported a relation between rapid gastric emptying and postprandial symptoms such as nausea, diarrhea, sweating and retching. One study [20] evaluated the use of enteral formulas with high (2:1 v/v) or low pectin (3:1 v/v) for 4 weeks, finding a significant reduction in GER compared to a formula with no pectin (**Table 1**).

Two studies investigated the effect of supplementation with vitamin D on the plasma concentration of 25-hydroxyvitamin D, with a total of 63 participants. Age varied between 6 and 18 years old. The study of LeRoy et al. (2015) evaluated a single oral dose of 100,000 UI compared to a placebo [7]. Kilpinen-Loisa et al. (2007) investigated the dose of 1,000 UI, 5 days/week orally for 10 weeks compared to an observational group (no placebo) [24]. Both studies found improvements in 25-hydroxy-vitamin D plasma concentrations for children receiving intervention compared to control groups. Moreover, the interventions were not associated with hypercalcemia or any other adverse effects (**Table 2**). Besides the plasma concentration of 25-hydroxy-vitamin D, Kilpinen-Loisa et al (2007). also investigated levels of calcium, phosphate, parathyroid hormone and markers of bone formation and resorption (alkaline phosphatase, serum aminoterminal propeptide of type I procollagen and telopeptide of type I collagen). The authors did not find significant differences in these markers of bone metabolism between groups who received vitamin D supplementation or not.

Two studies evaluated anthropometric measures and nutritional status as the main outcomes, comprising 24 participants. One study included only children below 5 years of age, and the other had a sample composed of individuals between 2.8 and 15.8 years old. Sevilla Paz Soldán et al. (2018)

investigated supplementation with a lipid mixture (coconut 35%, olive 35%, fish 15%, soy 15%) for 6 months [11]. Patrick et al. (1986) evaluated a diet with high energy density for 5 weeks [18]. Both interventions resulted in significant improvement of anthropometric measures, such as weight, arm circumference and triceps skinfold. Moreover, the lipid mixture was associated with a better lipid profile and psychomotor development at the end of the intervention compared to the control group (**Table 3**).

Two studies applied interventions to improve constipation and stool characteristics. Age ranged between 1 and 12 years old for the 136 participants of both studies. García-Contreras et al. (2020) [10] evaluated supplementation for 28 days with probiotics, symbiotics or prebiotics compared to a placebo. Hassanein et al. (2021) [25] studied supplementation with an oral solution of magnesium sulfate for 1 month compared to a placebo. Both studies found positive results of their interventions, with improvements in stool consistency and frequency (**Table 4**).

Three studies evaluated other outcomes, comprising gross motor function, iron deficiency anemia and feeding skills. Aiming to improve gross motor function, Leal-Martinez et al (2020) [26] compared a nutritional support system with the diet recommended by the World Health Organization (WHO) and an observational group of patients between 4 and 12 years old. The authors observed a superior motor function performance of the intervention group after 7 and 13 weeks, especially for the standing and walking parameters (**Table 5**).

Omar et al. (2021) [27] studied lactoferrin versus iron hydroxide polymaltose complex for the treatment of iron deficiency anemia. Children between 1 and 10 years old received the interventions for 4 weeks. ~~Laboratorial markers~~ of anemia were improved in both groups, but the variation in hemoglobin and ferritin was greater in the lactoferrin group. Additionally, children in the lactoferrin group presented a lower incidence of constipation as an adverse effect (**Table 5**).

The study of Mlinda et al. (2018) [28] investigated whether an educational intervention could improve feeding skills. The intervention consisted of group and individual nutritional education,

training of caregivers on positioning during feeding and occupational therapy. Caregivers in the intervention group reported significant improvements in skills, positioning, feeding speed, child involvement during feeding and child-caregiver interaction, in addition to less stress and better child mood during feeding (**Table 5**).

The results must be interpreted with caution since the overall risk of bias was high for the majority of studies (60.0%). The remaining studies were evaluated with some concerns (30.0%), with no studies presenting low risk of bias. More than 40% of studies presented a high risk of bias or some concerns for three of the five domains evaluated: bias arising from the randomization process (13.3% high; 33.3% some concerns), bias due to deviations from intended interventions (6.7% high; 53.3% some concerns), and bias in selection of the reported results (100% some concerns) (**Fig 2A**).

Ten of the 15 studies (66.7%) did not present a high risk of bias in any domain. Four studies presented some concerns only in one domain (bias in selection of the reported results) [7,12,20,22]; two presented some concerns in two domains [21,27]; and four presented some concerns in three domains [11,19,25,28]. In the five studies with high-risk domains, three presented high risk in only one domain [18,24,26], and two studies presented high risk of bias for two domains [10] (**Fig 2B**).

Figure 2. Risk of bias assessment using Cochrane tool for assessing risk of bias in randomized trials. A: Risk of bias according to selected domains for each study; B: Proportion of studies with low, high, and unclear risk of bias for each selected domain.

Discussion

The present study synthesized the results and evaluated the risk of bias in studies investigating diet and nutritional interventions on clinical, nutritional and neurodevelopment outcomes in children with CP. Interventions and outcomes varied between the included studies and can be summarized as follows: whey-based or pectin enriched enteral formulas for gastroesophageal reflux; 25-hydroxy-

vitamin D supplementation for hypovitaminosis D; supplementation with lipid mixture or diet with high density energy for improvements in anthropometric measures; supplementation with probiotics, prebiotics, symbiotics or magnesium for constipation; nutritional support system for gross-motor function; lactoferrin or iron hydroxide polymaltose for iron deficiency anemia; and educational intervention to improve feeding skills. All studies found positive results and did not report any important adverse outcome. However, evidence is weak since few studies were performed, and the risk of bias is high for the majority of performed studies.

[illegible]is is the first SLR specifically regarding dietary and nutritional interventions in children with CP. We searched for a broad spectrum of interventions and outcomes in addition to evaluating the risk of bias, which makes this SLR a good referential for health professionals regarding evidence-based practice. Moreover, our search was very sensitive and included gray literature databases, minimizing publication bias. Unfortunately, the results did not allow us to perform a meta-analysis for any intervention/outcome, as the included studies were very heterogeneous.

In the studies investigating gastric emptying as an outcome, the proportion of participants with fundoplication was diverse. Included studies with a greater proportion of participants with fundoplication were also those observing more side effects related to faster gastric emptying. Fundoplication is an anti-reflux surgery known for accelerating gastric emptying and may cause dumping syndrome as an adverse effect [29,30]. Therefore, faster gastric emptying in children with fundoplication may be an undesirable outcome, leading to gastrointestinal symptoms, such as gagging, regurgitation, irritability, and pain.

The two studies that evaluated supplementation with vitamin D were performed in cities with low sunlight incidence, Santiago (Chile) and Helsinki (Finland), with latitudes of -33.5 and 60.2, respectively [7,24]. Whereas the closer to zero latitude, the greater the incidence of sunlight, the dosage of vitamin D supplementation to achieve adequate plasma concentrations may vary according to geographical location, being lower for locations near the equator [31].

Regarding the use of magnesium sulfate as an intervention for constipation, the authors raised the limitation that safety data after prolonged treatment for one year were not evaluated [25]. Hypermagnesemia (values above 4 mg/dL) may lead to weakness, nausea, dizziness, confusion, decreased reflexes, worsening confusional state, drowsiness, bladder paralysis, flushing, headache and constipation. Values above 12 mg/dL may cause cardiovascular complications and neurological disorders. Values above 15 mg/dL can induce cardiorespiratory arrest and coma [32]. Therefore, the use of this intervention, especially for long periods, must require close monitoring of magnesium serum levels. Additionally, it may not be recommended for patients with renal disorders, since renal function is crucial for magnesium metabolism [33].

The domains judged as high risk of bias are the most likely cause of spurious associations. The randomization process represented a high risk of bias for two studies. In the study of Kilpinen-Loisa et al. (2007) [24], the allocation sequence was not considered random. The allocation was dependent on the baseline serum concentrations of vitamin D that were arranged in increasing order, and every other child was assigned to receive the supplement. In turn, Leal-Martinez et al. (2020) [26] performed adequate randomization, but baseline results of the motor function scale showed an important difference between groups. Although differences were not statistically significant, the lack of significance may be merely due to the small sample size. They performed an analysis by percentage of evolution to overcome this limitation, but it is still a concern and may be overestimating the effect.

Garcia-Contreras et al. (2020) [10] had a high risk of bias both due to deviations from the intended intervention and due to missing outcome data. Only 75% of the randomized subjects completed the intervention and were included in the analysis. Moreover, they did not use an appropriate analysis to estimate the effect of assignment to intervention. Patrick et al. (1986) [18] presented a high risk of bias arising from measurement of the outcome. The publication does not present information regarding the blindness of the assessors to the intervention status.

Graham-Parker et al. (2001) [23] is published as an abstract and, therefore, is missing many important information. It had a high risk of bias for two domains: missing outcome data and

measurement of outcome. Abstracts usually do not present information regarding losses of follow-up, consequently we were not able to determine how possible missing outcome data could affect the results. The measurement of outcomes was based on parents' or caregivers' records, which was not considered a reliable method.

Some concerns were raised in all the included studies regarding the bias in selection of the reported results. This occurred because most studies did not present a prespecified analysis plan. To evaluate this domain, the clinical trial register was consulted, and only the study of Garcia-Contreras et al. (2020) [10] did write an analysis plan, but it was not completely followed. Four studies presented some concerns in the overall analysis, instead of low risk, only because of this flaw [7,12,20,22].

None of the included studies performed an intention-to-treat analysis, which is also a major concern to the validity of the results and effectiveness of the interventions in clinical practice. Excluding participants from the analysis according to their adherence to the intervention may leave those who are meant to have a better outcome and damage the unbiased comparison given by randomization [34].

The small number of randomized clinical trials regarding the use of nutritional interventions in children with cerebral palsy together with the promising results found reveals a neglected area with the potential to improve health quality of life for millions of families around the world. Future studies must confirm the results of the interventions evaluated by previous authors using rigorous methods to obtain valid results and, hence, high-quality evidence. Special care must be taken in the randomization process, the intention-to-treat analysis and elaboration and publication of a prespecified analysis plan.

References

1. Surveillance of Cerebral Palsy in Europe. Surveillance of cerebral palsy in Europe: a collaboration of cerebral palsy surveys and registers. Surveillance of Cerebral Palsy in Europe (SCPE). Dev Med Child Neurol. 2000;42: 816–824. doi:10.1017/s0012162200001511

2. Rosenbaum P, Paneth N, Leviton A, Goldstein M, Bax M, Damiano D, et al. A report: the definition and classification of cerebral palsy April 2006. Dev Med Child Neurol Suppl. 2007;109: 8–14.

3. Ruiz Brunner M de LM, Cieri ME, Rodriguez Marco MP, Schroeder AS, Cuestas E. Nutritional status of children with cerebral palsy attending rehabilitation centers. Dev Med Child Neurol. 2020;62: 1383–1388. doi:10.1111/dmcn.14667

4. Melunovic M, Hadzagic-Catibusic F, Bilalovic V, Rahmanovic S, Dizdar S. Anthropometric Parameters of Nutritional Status in Children with Cerebral Palsy. Mater Sociomed. 2017;29: 68–72. doi:10.5455/msm.2017.29.68-72

5. Araújo LA, Silva LR, Mendes FAA. Digestive tract neural control and gastrointestinal disorders in cerebral palsy. J Pediatr (Rio J). 2012;88: 455–464. doi:10.2223/JPED.2241

6. Sousa KT de, Ferreira GB, Santos AT, Nomelini QSS, Minussi LO de A, Rezende ÉRM de A, et al. Assessment of nutritional status and frequency of complications associated to feeding in patients with spastic quadriplegic cerebral palsy. Rev Paul Pediatr. 2020;38: e2018410. doi:10.1590/1984-0462/2020/38/2018410

7. Le Roy C, Meier M, Witting S, Pérez-Bravo F, Solano C, Castillo-Durán C. [Effect of supplementation with a single dose of vitamin D in children with cerebral palsy. Preliminary randomised controlled study]. Rev Chil Pediatr. 2015;86: 393–398. doi:10.1016/j.rchipe.2015.07.015

8. Bhatia J, Griffin I, Anderson D, Kler N, Domellöf M. Selected macro/micronutrient needs of the routine preterm infant. J Pediatr. 2013;162: S48-55. doi:10.1016/j.jpeds.2012.11.053

9. King JC, Brown KH, Gibson RS, Krebs NF, Lowe NM, Siekmann JH, et al. Biomarkers of Nutrition for Development (BOND)-Zinc Review. J Nutr. 2016. doi:10.3945/jn.115.220079

10. Garcia-Contreras AA, Vasquez-Garibay EM, Sanchez-Ramirez CA, Fafutis-Morris M, Delgado-Rizo V. Lactobacillus reuteri DSM 17938 and Agave Inulin in Children with Cerebral Palsy and Chronic Constipation: A Double-Blind Randomized Placebo Controlled Clinical Trial. NUTRIENTS. ST ALBAN-ANLAGE 66, CH-4052 BASEL, SWITZERLAND: MDPI; 2020. doi:10.3390/nu12102971

11. Sevilla Paz Soldán R, Condori Bustillos R, Sejas Claros A, Huayhua Mexicano R, Calla Domaire P. Mezcla lipidica para mejorar el desarrollo psicomotriz en niños menores de 5 años con paralisis cerebral infantil^ies. Rev cientif cienc med. 2018;21: 21–28.

12. Brun AC, Størdal K, Johannesdottir GB, Bentsen BS, Medhus AW. The effect of protein composition in liquid meals on gastric emptying rate in children with cerebral palsy. Clin Nutr. 2012;31: 108–112. doi:10.1016/j.clnu.2011.07.009

13. Cochrane Collaboration. Cochrane handbook for systematic reviews of interventions. Chichester, England ; Hoboken, NJ: Wiley-Blackwell; 2008.

14. Page MJ, McKenzie J, Bossuyt P, Boutron I, Hoffmann T, Mulrow C, et al. The PRISMA 2020 statement: an updated guideline for reporting systematic reviews. MetaArXiv; 2020. doi:10.31222/osf.io/v7gm2

15. Covidence systematic review software. Melbourne, Australia: Veritas Health Innovation; Available: www.covidence.org

16. Sterne JAC, Savović J, Page MJ, Elbers RG, Blencowe NS, Boutron I, et al. RoB 2: a revised tool for assessing risk of bias in randomised trials. BMJ. 2019;366: l4898. doi:10.1136/bmj.l4898

17. McGuinness LA, Higgins JPT. Risk-of-bias VISualization (robvis): An R package and Shiny web app for visualizing risk-of-bias assessments. Res Synth Methods. 2021;12: 55–61. doi:10.1002/jrsm.1411

18. Patrick J, Boland M, Stoski D, Murray GE. Rapid correction of wasting in children with cerebral palsy. Dev Med Child Neurol. 1986;28: 734–739. doi:10.1111/j.1469-8749.1986.tb03925.x

19. Khoshoo V, Zembo M, King A, Dhar M, Reifen R, Pencharz P. Incidence of gastroesophageal reflux with whey- and casein-based formulas in infants and in children with severe neurological impairment. J Pediatr Gastroenterol Nutr. 1996;22: 48–55. doi:10.1097/00005176-199601000-00008

20. Miyazawa R, Tomomasa T, Kaneko H, Arakawa H, Shimizu N, Morikawa A. Effects of pectin liquid on gastroesophageal reflux disease in children with cerebral palsy. BMC Gastroenterol. 2008;8: 11. doi:10.1186/1471-230X-8-11

21. Savage K, Kritas S, Schwarzer A, Davidson G, Omari T. Whey- vs casein-based enteral formula and gastrointestinal function in children with cerebral palsy. JPEN J Parenter Enteral Nutr. 2012;36: 118S–23S. doi:10.1177/0148607111428139

22. Fried MD, Khoshoo V, Seeke DJ, Gilday DL, Ash JM, Peneharz PB. Decrease in gastric emptying time and episodes of regurgitation in children with spastic quadriplegia fed a whey-based formula. The Journal of Pediatrics. 1992;120: 569–572. doi:10.1016/S0022-3476(10)80003-4

23. Graham-Parker C. Effect of whey-based diet on incidence of vomiting in G-tube fed children with severe neurodevelopmental delay. JPEN J Parenter Enteral Nutr. 2001;25: S14.

24. Kilpinen-Loisa P, Nenonen H, Pihko H, Mäkitie O. High-dose vitamin D supplementation in children with cerebral palsy or neuromuscular disorder. Neuropediatrics. 2007;38: 167–172. doi:10.1055/s-2007-990266

25. Hassanein SMA, Deifallah SM, Bastawy HA. Efficacy of oral magnesium therapy in the treatment of chronic constipation in spastic cerebral palsy children: a randomized controlled trial. World J Pediatr. 2021;17: 92–98. doi:10.1007/s12519-020-00401-0

26. Leal-Martínez F, Franco D, Peña-Ruiz A, Castro-Silva F, Escudero-Espinosa AA, Rolón-Lacarrier OG, et al. Effect of a Nutritional Support System (Diet and Supplements) for Improving Gross Motor Function in Cerebral Palsy: An Exploratory Randomized Controlled Clinical Trial. Foods. 2020;9. doi:10.3390/foods9101449

27. Omar OM, Assem H, Ahmed D, Abd Elmaksoud MS. Lactoferrin versus iron hydroxide polymaltose complex for the treatment of iron deficiency anemia in children with cerebral palsy: a randomized controlled trial. Eur J Pediatr. 2021;180: 2609–2618. doi:10.1007/s00431-021-04125-9

28. Mlinda SJ, Leyna GH, Massawe A. The effect of a practical nutrition education programme on feeding skills of caregivers of children with cerebral palsy at Muhimbili National Hospital, in Tanzania. Child Care Health Dev. 2018;44: 452–461. doi:10.1111/cch.12553

29. Estevão-Costa J, Fragoso AC, Prata MJ, Campos M, Trindade E, Dias JA, et al. Gastric emptying and antireflux surgery. Pediatr Surg Int. 2011;27: 367–371. doi:10.1007/s00383-010-2702-6

30. Yamoto M, Fukumoto K, Takahashi T, Miyake H, Nakaya K, Nomura A, et al. Risk factors of dumping syndrome after fundoplication for gastroesophageal reflux in children. Pediatr Surg Int. 2021;37: 183–189. doi:10.1007/s00383-020-04783-0

31. Mendes MM, Hart KH, Williams EL, Mendis J, Lanham-New SA, Botelho PB. Vitamin D Supplementation and Sunlight Exposure on Serum Vitamin D Concentrations in 2 Parallel, Double-Blind, Randomized, Placebo-Controlled Trials. J Nutr. 2021; nxab209. doi:10.1093/jn/nxab209

32. Cascella M, Vaqar S. Hypermagnesemia. StatPearls. Treasure Island (FL): StatPearls Publishing; 2021. Available: http://www.ncbi.nlm.nih.gov/books/NBK549811/

33. Seo JW, Park TJ. Magnesium Metabolism. Electrolyte Blood Press. 2008;6: 86–95. doi:10.5049/EBP.2008.6.2.86

34. Montori VM, Guyatt GH. Intention-to-treat principle. CMAJ. 2001;165: 1339–1341.

Supporting information captions

S1 Appendix. Search summary: databases, search strategies and number of identified records.

S2 Appendix. PROSPERO Register details.

S3 Appendix. PRISMA Checklist.

S1 Table. Detailed risk of bias assessment using Cochrane tool for assessing risk of bias in randomized trials (RoB 2).

S2 Table. References excluded after full-text evaluation and respective reasons.

Figure 1

Click here to access/download;Figure;Figure 1_PRISMA flowchart.tif

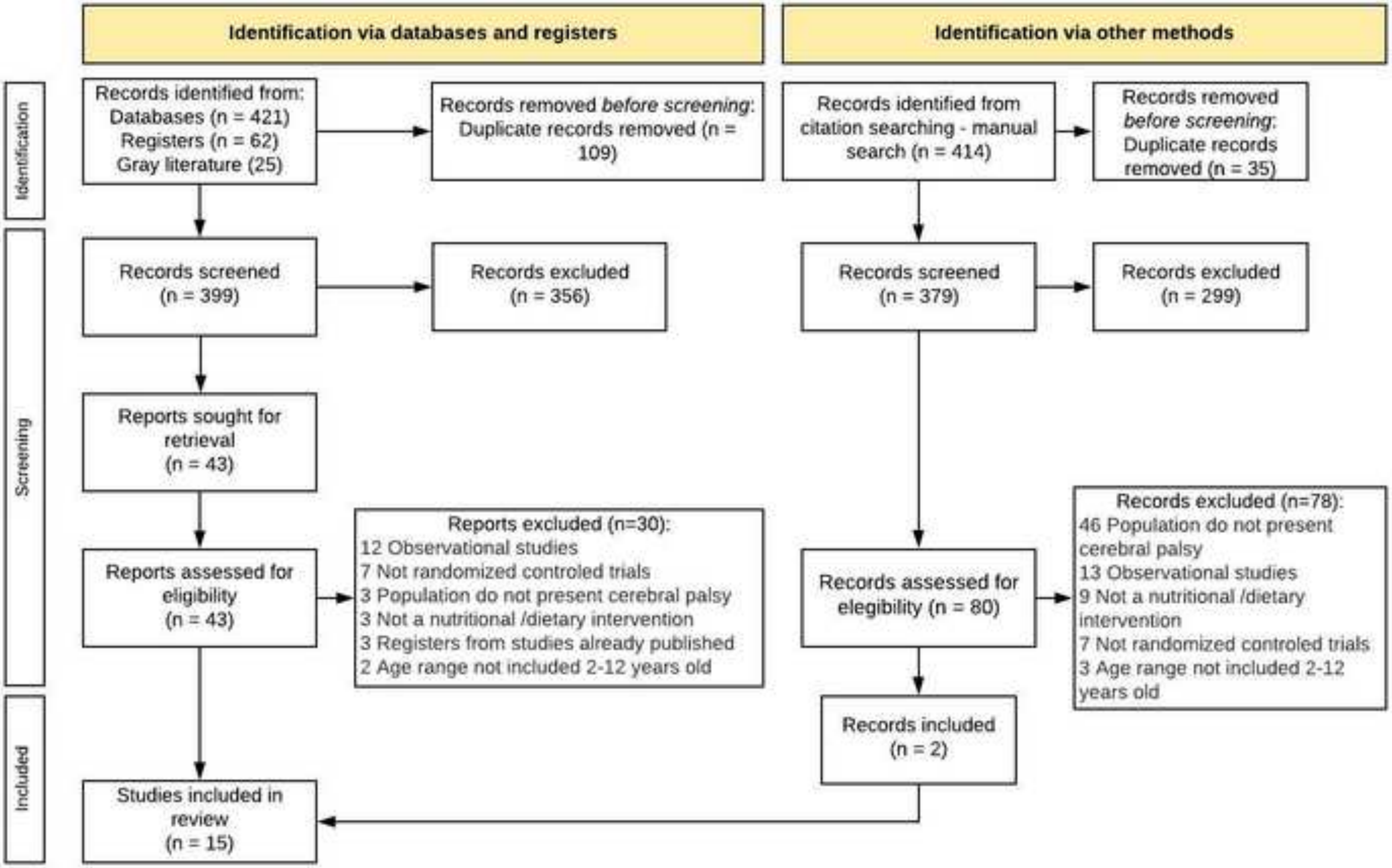

Figure 2

Click here to access/download;Figure;Figure 2_ROB 2-1.tif

A.

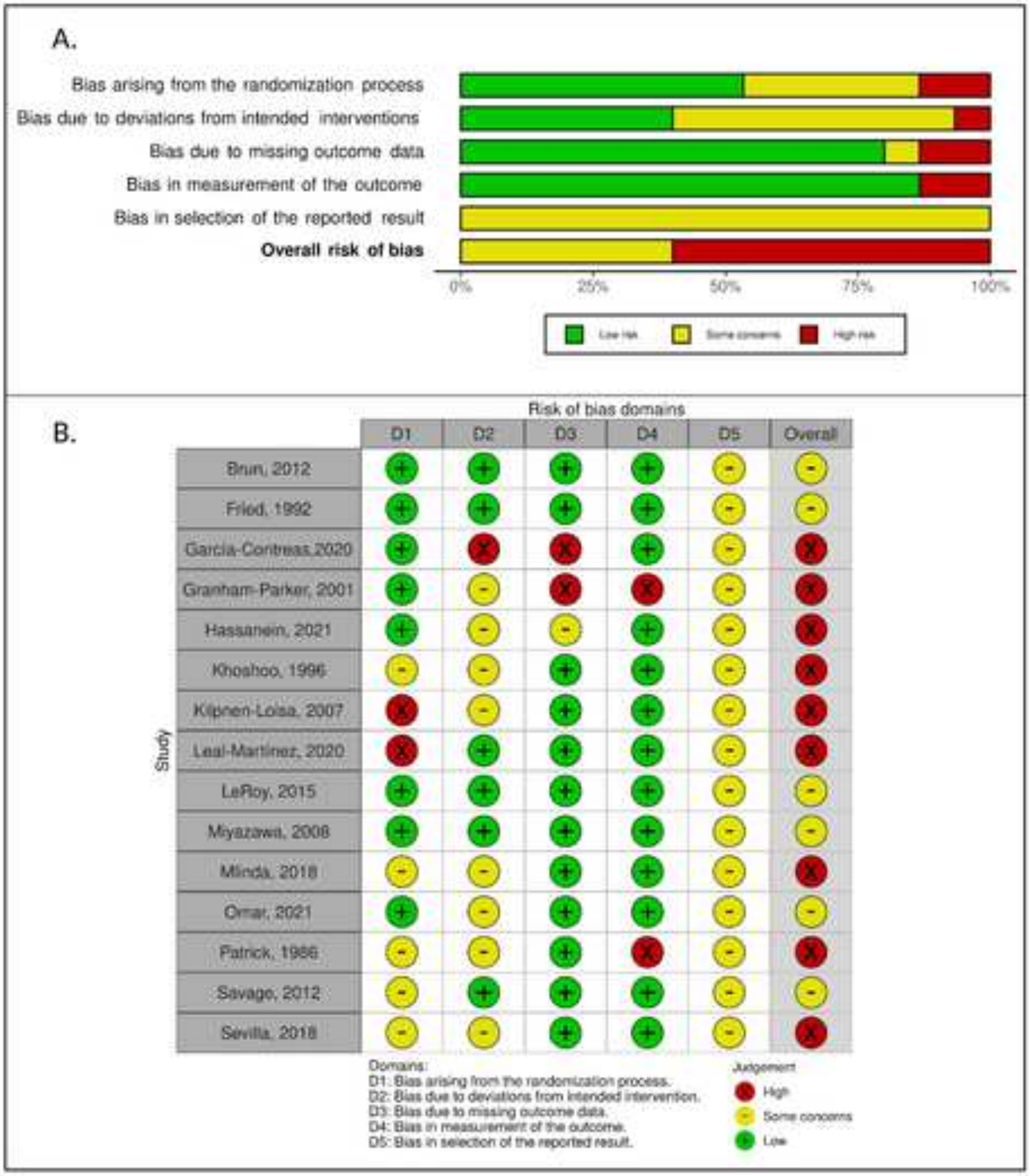

S1 Table

Click here to access/download
Supporting Information
S1 Table.xlsx

S2 Table

Click here to access/download
Supporting Information
S2 Table.xlsx

Click here to access/download
Supporting Information
S1 Appendix.docx

S2 Appendix - PROSPERO Registration

Click here to access/download
Supporting Information
S2 Appendix_PROSPERO.pdf

S3 Appendix - PRISMA Checklist

Click here to access/download
Supporting Information
S3 Appendix.docx
